# Pre‐pregnancy iodized salt improved children's cognitive development in randomized trial in Ethiopia

**DOI:** 10.1111/mcn.12943

**Published:** 2020-01-07

**Authors:** Husein Mohammed, Grace S. Marquis, Frances Aboud, Karim Bougma, Aregash Samuel

**Affiliations:** ^1^ Nutrition and Food Science Department University of Ghana Accra Ghana; ^2^ School of Dietetics and Human Nutrition McGill University Quebec Canada; ^3^ Department of Psychology McGill University Quebec Canada; ^4^ Food Sciences and Nutrition Research Directorate Ethiopian Public Health Institute Addis Ababa Ethiopia

**Keywords:** child mental development, iodine deficiency, iodized salt, pregnant women

## Abstract

The overarching Ethiopia project examined the effects of early market introduction of iodized salt on the growth and mental development of young children. Sixty districts were randomly assigned to intervention (early market access to iodized salt) or control (later access through market forces), and one community per district was randomly chosen as the sampling unit. For this project, 22 of the districts were included. The participants were 1,220 pregnant women who conceived after the intervention began. When their children were 2 to 13 months old, field staff collected information on household sociodemographic status and iodized salt intake, child stimulation, maternal depression symptoms, children's diet, anthropometry, urinary iodine concentration (UIC), hemoglobin, and mental development scores (Bayley III scales). Fewer mothers prepartum (28% vs. 41%, *p* < .05) and their children (13% vs. 20%, *p* < .05) were iodine deficient (UIC <50 μg/L) in the intervention compared with the control group. The intervention children had higher cognitive scores (33.3 ± 0.3 vs. 32.6 ± 0.3; Δ = 0.6; 95% CI [0.0, 1.3]; *d* = 0.17; *p* = .01; 4 IQ points) than their controls. The other Bayley subscale scores did not differ from control children. The intervention group had a higher child stimulation (22.7 ± 0.2 vs. 22.1 ± 0.2; Δ = 0.5; 95% CI [0.02, 0.89]; *d* = 0.17; *p* = .01) but not growth indicators (weight‐for‐age *z* score, length‐for‐age *z* score, and weight‐for‐length *z* score: −1.1 ± 0.1 vs. −1.1 ± 0.1, −1.7 ± 0.1 vs. −1.7 ± 0.1; −0.2 ± 0.1 vs. −0.1 ± 0.1, respectively, all *p* > .05) compared with their controls. Iodized salt intake improved iodine status of both pregnant women and their children and also child cognitive development.

**Key messages**

- Children have better cognitive development when exposed to iodized salt from neonatal stage to childhood.
- If implemented well,the USI policy can meet the iodine requirement of a population including those of the vulnerable group (pregnant women, infants, and children).
- RCTs are needed to provide solid evidence on policies and practices that reduces the prevalence of ID in populations around the world.

## 1 INTRODUCTION

Iodine deficiency (ID) is a major public health problem for populations throughout the world with a global incidence of 29% (Andersson, Karumbunathan, & Zimmermann, 2012), and it is the main cause of preventable mental retardation in childhood (Delange et al., 1993; Hetzel, 2000). Iodine is required for the synthesis of thyroid hormones that is necessary for brain development especially at the early stages of life. Supplementation studies of pregnant women have largely found the same effect size of 0.48 for child mental development, regardless of design (Bougma, Aboud, Harding, & Marquis, 2013). Only two supplementation studies in Zaire and Peru used a randomized clinical trial design (Pretell, Torres, Zenteno, & Cornejo, 1972; Thilly, 1981). The study in Zaire found positive effects, whereas the Peruvian study found no significant effect of iodine supplementation in pregnancy on child mental development. No previous randomized clinical trial has used iodized salt as a means to enhance iodine sufficiency in pregnant women or children (Aburto, Abudou, Candeias, & Wu, 2014; Qian et al., 2005). Consequently, there is a need to examine with a rigorous design the effects of iodized salt given to mothers and infants on the mental and physical development of young children.

Recent surveys conducted in Ethiopia revealed a high prevalence of goiter averaging 40% and correspondingly low levels of urinary iodine (UI) excretion (median 24.5 μg/L; Abuye, Berhane, Akalu, Getahun, & Ersumo, 2007). Iodized salt, the most common strategy for improving iodine consumption in low‐iodine regions, had not been commonly available in Ethiopia for several decades (Abuye et al., 2007; Abuye, Berhane, & Ersumo, 2008). The Ethiopian government passed legislation on universal salt iodization in 2011 to make iodized salt gradually available to families through the open market. This provided an opportunity to use a randomized design to answer questions concerning the effect of iodized salt exposure from pregnancy to early childhood on infants' physical and mental development. For practical reasons, we adopted a cluster randomized design to minimize treatment contamination and use the existing district‐based distribution of salt to markets. It was hypothesized that children whose mothers were living in districts with early introduction of iodized salt would have higher mental and physical development than those in control districts.

## 2 METHODS

### 2.1 Research area, design, and participants

**Figure 1:**
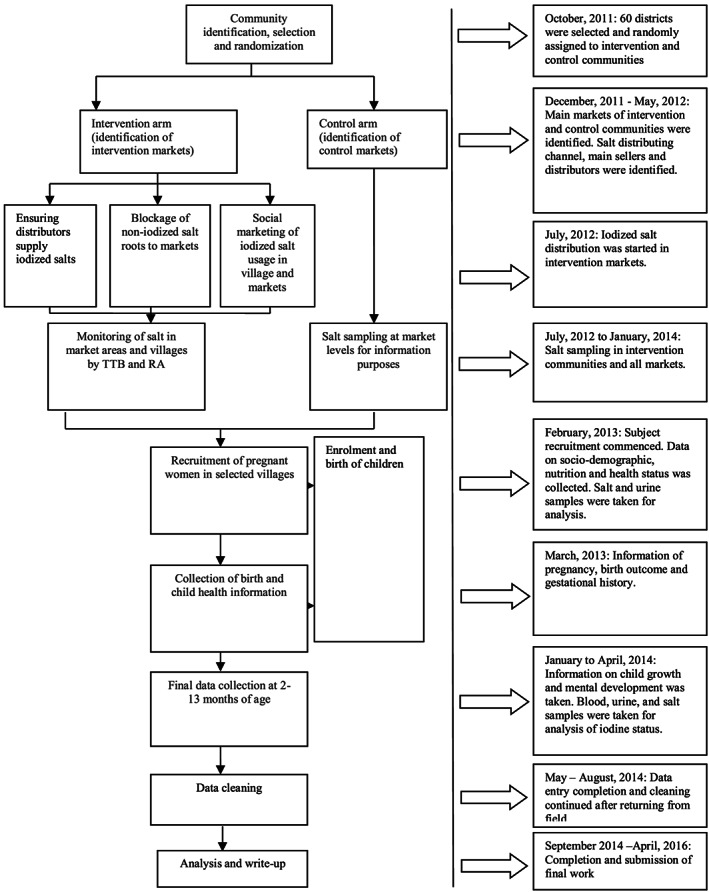
Study design and time line

**Figure 2:**
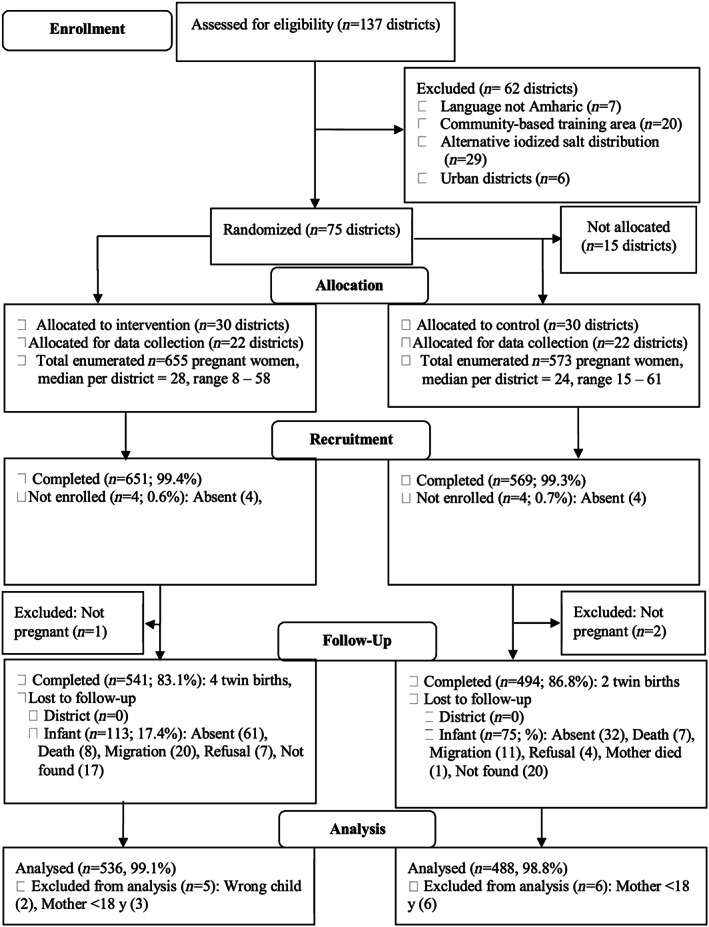
Consolidated Standards of Reporting Trials 2010 flow diagram

The research design was a cluster randomized controlled trial, consisting of 60 randomly selected districts (out of 71 existing Amharic‐speaking districts) within 6 of 10 contiguous zones in the Amhara Region of Ethiopia. The intervention was randomized to a cluster (district), the smallest administrative unit from which salt distribution was organized. A village was then randomly selected per district in 22 out of the 30 intervention and 22 out of 30 control districts for data collection of pregnant mothers and their offspring (Figures 1 and 2). Project principal investigators used computer generated random numbers for the randomization and allocation of intervention and control communities. Research assistants who participated in the data collection and study participants were blinded with respect to which communities were intervention and control. Salt monitoring personnel who were separate from data collection teams, monitored salt quality in the communities. Participants were women who became pregnant no earlier than 1 month after the early distribution of iodized salt into intervention communities began. As part of a census, local recruiters identified all pregnant women (starting at 2–3 months gestation). Inclusion criteria ensured that the selected women (a) had not received iodine capsules in the past year, (b) spoke Amharic, (c) were 18 years or older, (d) lived in the selected village for at least a year, and (e) planned to continue living in the selected village for at least another year (Figure 1).

### 2.2 Sample size determination

Two previous intervention trials with pregnant women in moderate ID areas gave an effect size between 0.02 and 1.38 (95% CI [−0.45, 2.32]) for mental development in children (Berbel et al., 2009; Velasco et al., 2009). This study's group sample size was calculated with a small effect size (*d*) of 0.2 and power of 0.8 using the formula: *n* = [2 (*Z*_α/2_ + *Z*_β_)^2^]/*d* ^2^; where *n* = number per group, *α* = 0.05, β = 0.2, *Z*_α/2_ = 1.96 and *Z*_β_ = 0.84 (Lenth, 2001). The sample size was 393 per group. The intracluster correlation was assumed to be 0.04. So the variance inflation factor (VIF) = 1 + (cluster size − 1) × intracluster correlation (Hutton, 2001). From a preliminary study, the average number of pregnant women per village was 30. A conservative cluster size of 18 was used, then accounting for the cluster: VIF = 1 + [(18 − 1) × 0.04] = 1.68. Hence, the adjusted sample size per group was 393 × 1.68 = 660 per group. The minimum number of clusters was therefore 660/30 = 22 clusters per group.

### 2.3 Intervention

In Ethiopia, salt is mainly produced in the Afar region from Lake Afdera and distributed in the Amhara Region by one of two regional distributors before going into the open market. The main distributors were contacted by the project to supply iodized salt from the Afar region to selected intervention markets as shown in Figure 1. The staffs of the district salt monitoring offices within each district were requested to monitor and stop the movement of noniodized salts into intervention markets and villages. The officers were trained and provided with rapid test kits (MBI Kits International, Chennai, Tamil Nadu, India) for monitoring. A project salt monitoring personnel (who was not part of the data collection team) was also trained to monitor the quality of salt supplied by salt distributors and those sold within the market. Iodized salt in control markets and villages was left to market forces but sampled and tested for information purposes.

### 2.4 Outcome measures and data collection

#### 2.4.1 Assessment of pregnant women

At recruitment, data were collected on the goiter status of the pregnant women using the palpation method (World Health Organization [WHO], 2001). Urine samples of the pregnant women were also taken in urine tubes in a nonfasting state. The urine samples were preserved with 29.2 g/L boric acid (Meers & Chow, 1990), sealed with paraffin film, protected from sunlight, and stored in the village health office at room temperature for up to 1 week. Urine samples were subsequently frozen at −20°C and analyzed at the Ethiopian Public Health Institute (EPHI) by modified Sandell–Kolthoff reaction (Jooste & Strydom, 2010). For only a subsample of women who were present and still pregnant during a field visit after their recruitment, hemoglobin was collected using a HemoCueTM 201+ portable photometer (Sanchis‐Gomar, Cortell‐Ballester, Pareja‐Galeano, Banfi, & Lippi, 2013).

#### 2.4.2 Post‐partum measures and data collection

The primary outcome measure was Bayley Scales of Infant and Toddler Development III (Bayley, 2006). The Bayley tests were administered at home when children were between 2 and 13 months old. Four subscales of the Bayley were used, namely, cognitive (91 items), expressive language (48 items), receptive language (49 items), and fine motor (66 items). The scales have been previously used in Ethiopia (Aboud & Alemu, 1995; Asefa, Kaba, Tessema, Drewett, & Wolke, 2001) and other low‐ to middle‐income countries such as Bangladesh (Black et al., 2004) and Kenya (Sigman et al., 1988). Research assistants conducted interviews with the mother to elicit household information, namely, sociodemographic characteristics such as assets (10 household assets), and goiter in the family; household food security was assessed with the Household Food Insecurity Access Scale (Coates, Swindale, & Bilinsky, 2007). Information about the mother included depression symptoms using the 60‐item Centre for Epidemiological Studies Depression scale (<20 out of 60 was nondepressed; Radloff, 1977), gestational history, and a 24‐hr food frequency interview that was translated into a dietary diversity (score 0 to 7), along with directly measured maternal weight and height.

Mothers reported information about the child, including the following: dietary intake (breastfeeding and dietary diversity of children over 6 months old), symptoms of illness in the past 14 days, and immunization status. Secondary outcomes such as gross motor milestones were assessed using the WHO's motor development milestones (Group & de Onis, 2006), psychosocial stimulation of infants was assessed using the 45‐item Home Observation for Measurement of the Environment (HOME) inventory (Bradley, Corwyn, & Whiteside‐Mansell, 1996), and a child hygiene score (13‐item environmental cleanliness and hygiene practices around child). Data were collected on children's anthropometry (weight, height, and midupper arm circumference) in duplicate by two research assistants. The goiter status was assessed by palpation (WHO, 2001), and hemoglobin was tested by the azide‐methemoglobin method with a HemoCue^TM^ 201+ portable photometer (Sanchis‐Gomar et al., 2013). Anemia was defined as hemoglobin less than 110 mg/dl. Infant urine samples were collected in urine tubes, frozen at −20°C and sent to EPHI to analyze UI concentration. The urine was stored at −20°C prior to analysis. Household salt samples were taken and checked for iodine using the rapid test kits (WHO, 2001). Twenty percent of collected household salt that tested positive for iodine were sent to the laboratory at EPHI together with the urine samples to analyze iodine levels, using the modified Sandell–Kolthoff reaction (Jooste & Strydom, 2010). There were no reported adverse effects of the intervention on the participants or their communities.

### 2.5 Data analysis

The Bayley raw scores were standardized by age using the transformations provided by the Bayley administration manual (Bayley, 2006). Responses to HOME and Centre for Epidemiological Studies Depression completed questionnaires were tallied. Group differences of the sociodemographic characteristics were analyzed in order to identify covariates. The treatment effects on the mental and physical development scores were examined with SAS 9.4 PROC MIXED (for continuous variables) and GLIMMIX (for proportions) adjusted for clusters and covariates from preplanned conventional covariates from the literature (child age, assets, and maternal education). Modifier effects on the treatment were assessed by including a modifier‐by‐treatment interaction term in the analysis of covariance (ANCOVA). Means were reported with standard deviations, and mixed models were reported with least‐squared means and standard errors.

### 2.6 Ethical statement

Ethical clearance was obtained from McGill University and EPHI and the Ethiopian National Research Ethics Review Committee. Written permission was obtained from the Regional Health Bureau of the Amhara Region, from the zone, district, and village offices, and all the Trade and Transport Bureau offices within the selected districts. Written informed consent forms were signed or thumb printed by mothers after thoroughly understanding the study. The study was registered at ClinicalTrials.gov with the registration number NCT01349634.

## 3 RESULTS

The baseline characteristics of the selected communities have been published earlier (Aboud, Bougma, Lemma, & Marquis, 2017; Bougma, Aboud, Lemma, Frongillo, & Marquis, 2018). At recruitment during pregnancy, a total of 1,220 respondents were recruited of whom 1,024 (84%) were included in this analysis (Figure 2). Mothers in the intervention compared with control communities recorded a significantly higher UIC, with a median UIC difference of 43 μg/L; fewer intervention mothers had low UIC (<50 μg/L) compared with controls (11% vs. 15%), and goiter rate was lower in the intervention mothers, though family goiter was still high. Nonetheless, over 90% of households in each group were currently using iodized salt with half using salt with 15 ppm or more. Hemoglobin was taken from over one quarter of women (*n* = 346), and the mean levels were adequate; there were few cases of anemia in pregnancy (Table 1). However, over half of the participating mothers reported having symptoms of anemia during pregnancy (intervention vs. control, 55% vs. 62%; *p* < .05). A few mothers reported taking iodine capsule during the May 2012 distribution (3% intervention vs. 8% control; *p* < .0001).

**Table 1:** Iodine and iron indicators in pregnancy, at least 4 months after intervention began

Variables	Intervention	*N*	Control	*N*	*F* value/*χ* 2	*P*
Household						
Household salt iodized, *n* (%)	556 (91.6)	607	510 (95.3)	535	6.4	.01
Salt iodine at least 15 ppm, *n* (%)	32(50.8)	63	20 (41.7)	48	0.91	0.34
Maternal						
UI (μg/L), median (IQR)	163.8 (93.2, 263.5)	645	120.6 (68.9, 216.4)	562	5.3	<.001
Low UI (<150 μg/L), *n* (%)	290 (45.0)	645	340 (60.5)	562	29.1	<.001
Goiter (any) ^1^, *n* (%)	103 (15.9)	647	117 (21.2)	553	5.5	.02
Hemoglobin (mg/dL), LSM (*SE*)	123.8 ± 1.1	201	130.0 ± 1.3	143	14.1	<.002
Anemia (<110 mg/dl) ^2^, *n* (%)	32 (15.9)	201	8 (5.6)	143	8.7	.003

*Note.* *n* (%) and *χ* 2 value; unless reported otherwise.

Abbreviations: IQR, interquartile range; LSM, least squared mean; *SE* = standard error; UI = urinary iodine.

1 Goiter = assessed by palpation method and categorized as yes or no (World Health Organization, 2001).

2 Anemia = Hemoglobin < 110 mg/dl.

At final data collection, the mothers were on the average 28 years of age, their mean body mass index was normal, and their mean dietary diversity score did not vary between the intervention and control groups and was above the minimum adequate level of four out of seven. However, their assets were low (2.4 out of 10), lack of any schooling was high (77%), and over 60% of households had water and sanitation practices that were poor (≤1 out of three), with the control group showing poorer water and sanitation practices. Most households used iodized salt (92%), but only half of the salt had iodine content ≥15 ppm. Eleven percent of the households were food insecure, which did not vary between the two groups. Data collection on the children was done at an average age of 8.1 months, and there was almost an even distribution of girls and boys (female vs. male, 47% vs. 53%). The intervention children had a significantly higher UI level (median 141 vs. 113 μg/L) than those in the control communities, but the levels for both communities were within the normal range. Fewer intervention than control children had UI less than 50 μg/L at the final data collection (*p* < .05). Mean hemoglobin levels of intervention children were lower (108 vs. 113 mg/dL, *p* < .0001), with a little over half of the intervention children and over a third of the control children anemic. Children's dietary diversity score of those older than 6 months of age was below the minimum adequate level (1.3 out of seven food groups) and did not vary between groups. Mothers reported that a little over a third of their children were sick (38%) in the 2 weeks preceding the final data collection.

Bayley scores of mental development, which are our main outcomes, were analyzed using a mixed ANCOVA model accounting for clusters and covariates, namely, child age, mother's education, and assets (Table 2). The cognitive scores of young children in the intervention communities were significantly higher than that of the control children, with an effect size of *d* = 0.17. Modifier analyses showed that the intervention children performed better on cognitive items than controls regardless of sex and nutritional status. There was no significant main effect on any of the other mental development scores. However, maternal depressive symptoms and home stimulation modified the effect of iodized salt exposure on several Bayley scores (Table 3). Intervention children performed better than controls on three indicators of mental development if their mothers reported low depressive symptoms (*d* = 0.22, 0.14, and 0.10 for cognitive, expressive, and receptive language, respectively). Additionally, HOME stimulation significantly modified the intervention effect on child's cognition such that children with lower home stimulation performed significantly better if they were in the intervention compared with control group (*d* = 0.44), whereas children with higher home stimulation performed better regardless. Maternal education also modified the effect of the intervention on expressive language such that children whose mothers had attended some schooling performed better than controls, whereas the effect of iodized salt was lost among children whose mothers had not attended school (effect size 0.39 vs. −0.05 in high vs. low levels of maternal education).

**Table 2:** Results of the PROC MIXED evaluating the effect of iodized salt on physical and mental development of children in Amhara Region of Ethiopia

Variable	Intervention		Control			Δ (95% CI)	ICC	Effect size, *d* (95% CI)
	*M* ± *SD*	*n*	*M* ± *SD*	*n*				
Maternal								
Urinary iodine (μg/L)	173.1 ± 25.0	410	132.1 ± 21.7	351		43.3 [26.9, 59.7]*	0.02	1.79 (1.57 to 1.90)*
Child								
Urinary iodine (μg/L)	161.4 ± 29.7	406	141.0 ± 31.9	362		21.0 [−3.2, 45.2]	0.02	0.89 (0.52 to 0.81)*
Hemoglobin (mg/dl)	107.6 ± 58.1	410	113.2 ± 61.4	350		−4.2 [−6.8, −1.5]*	0.09	−0.09 (−0.24 to 0.05)
WAZ	−1.2 ± 1.2	455	−1.2 ± 1.1	407		0.0 [−0.2, 0.2]	0.07	0.01(−0.13 to 0.13)
LAZ	−1.7 ± 1.1	455	−1.7 ± 1.2	402		0.1 [−0.1, 0.2]	0.16	0.06 (−0.13 to 0.13)
WLZ	−0.3 ± 1.2	455	−0.2 ± 1.1	406		−0.1 [−0.3, 0.0]	0.04	−0.10 (−0.22 to 0.05)
Bayley^1^								
Cognitive	33.0 ± 5.3	454	32.0 ± 5.2	410		0.6 [0.0, 1.3]*	0.01	0.17 (0.06 to 0.33)*
Cognitive—std.	9.9 ± 2.9	454	9.7 ± 3.0	410				
Receptive language	9.4 ± 1.8	454	9.3 ± 1.9	410		0.0 [−0.2, 0.3]	0.00	0.06 (−0.08 to 0.19)
Receptive language—std.	6.6 ± 2.2	454	6.6 ± 2.5	410				
Expressive language	10.7 ± 3.2	453	10.4 ± 3.2	409		0.1 [−0.2, 0.4]	0.04	0.05 (−0.04 to 0.23)
Expressive language—std.	9.9 ± 2.5	453	9.8 ± 2.7	409				
Fine motor	21.3 ± 3.0	453	21.1 ± 3.0	410		0.0 [−0.3, 0.4]	0.02	0.03 (−0.07 to 0.20)
Fine motor—std.	7.5 ± 2.6	453	7.8 ± 3.0	410				
Bayley—comp.	33.8 ± 7.9	454	33.8 ± 9.0	410		0.07 [−0.13, 0.13]	0.01	0.4 (−0.7 to 1.4)
Gross motor^2^	2.4 ± 1.6	407	2.3 ± 1.6	357		−0.03 [−0.08, 0.20]	0.10	−0.1 (−0.2 to 0.1)
							

*Note.* The covariates were child's age, maternal education, and assets.

Abbreviations: CI, confidence interval; comp., composite; ICC, intracluster correlation coefficient; LAZ, length‐for‐age *z* score; *SE*, standard error; WAZ weight‐for‐age *z* score; WLZ, weight‐for‐length *z* score, std., standardized.

* *p* < .05

1 Bayley scores, Bayley Scales of Infant and Toddler Development III (2006), reflects the competencies expected of infants and young children in the first 3.5 years. Four following scales are used: cognition (reasoning ability), receptive language (ability to understand language), expressive language (ability to express themselves), and fine motor (hand eye coordination). The Bayley composite score is the sum of the four standardized subscores (out of 80).

2 Gross motor, World Health Organization motor development competency of children; Mothers reported motor capabilities of their children, from 1 (sitting without support) to 7 (walking without support; Group & de Onis, 2006).

**Table 3:** Results of ANCOVA evaluating the interaction between the effect of iodized salt of mental development and their modifiers

Bayley scores^1^	Modifier	Modifier level (*n*)	Intervention, *M* (*SD*)	Control, *M* (*SD*)	Δ (95% CI)	Intervention, *F* (*p* value)	Modifier, *F* (*p* value)	Interaction, *F* (*p* value)	Effect size, *d* (95% CI)
Cognitive	Maternal depression symptoms^2^	Low (669)	33.2 (5.3)	31.9 (5.2)	1.6 [−0.2, 3.0]	0.2 [0.68]	0.1 [0.81]	5.5 [0.02]	+0.22 [0.09, 0.40]
		High (198; 183)	32.1 (5.3)	32.1 (5.2)	0.0 [0.0, 0.0]				−0.00 [−0.29, 0.29]
	HOME score^3^	Low(351)	31.5 (0.3)	29.5 (5.3)	1.7 [0.6, 2.7]	7.5 [<0.01]	49.8 [<0.0001]	13.8 [<0.001]	+0.44 [0.18, 0.60]
		High (501)	33.9 (0.2)	33.8 (4.3)	0.2 [−1.2, 0.8]				−0.07 [−0.16, 0.19]
								
Expressive language	Maternal depression symptoms	Low (668)	10.6 (3.2)	10.1 (3.2)	0.8 [0.0, 1.6]	0.3 [0.61]	15.0 [<0.0001]	4.7 [0.03]	+0.14 [0.02, 0.32]
		High (182)	10.9 (3.1)	10.9 (3.1)	0.0 [0.0, 0.0]				−0.03 [−0.32, 0.26]
								
	Maternal education	Low (649)	10.4 (3.2)	10.3 (3.2)	−1.1 [−1.9 to −0.3]	4.6 [0.03]	2.3 [0.13]	7.6 [<0.01]	+0.05 [−0.11, 0.20]
		High (201)	11.5 (3.0)	10.4 (3.1)	0.0 [0.0, 0.0]				+0.39 [0.08, 0.64]
								
Receptive language	Maternal depression symptoms	Low (669)	9.5 (1.8)	9.2 (1.9)	0.5 [0.0, 1.1]	0.5 [0.47]	2.2 [0.14]	3.5 [0.06]	+0.10 [−0.01, 0.29]
		High (183)	9.3 (1.7)	9.5 (1.8)	0.0 [0.0, 0.0]				+0.05 [−0.24, 0.34]

*Note.* Modifiers tested include maternal depression (high is ≥20), HOME score (high is ≥22), maternal education (low = no school), assets, water and sanitation, food security, and sex and growth indicators. Only significant interactions are presented here before running stratified analyses to obtain separate effect sizes.

1 Bayley scores, Bayley Scales of Infant and Toddler Development III (2006), reflects the competencies expected of infants and young children in the first 3.5 years. Four following scales are used: cognition (reasoning ability), receptive language (ability to understand language), expressive language (ability to express themselves), and fine motor (hand eye coordination). Sum of the standardized scores gives the Bayley standardized score, which is out of 80.

2 Maternal depression symptoms (Centre for Epidemiological Studies Depression [CES‐D]), a 20‐item measure of depressive symptoms (theoretic range 0–60; Radloff, 1977)

3 Home Observation for Measurement of Environment (HOME) score, a 45‐item interview and observation measures the amount and quality of stimulation and support provided to a child in the family setting (Bradley, 1996).

The prevalence of wasting (weight‐for‐length *z* score < −2 SD), underweight (weight‐for‐age *z* score < −2 SD), and stunting (length‐for‐age *z* score < −2 SD) were 5%, 23%, and 36%, respectively. A mixed ANCOVA accounting for clusters and covariates such as child age, mother's education, assets, child hygiene, and water and sanitation was used to analysis the anthropometric indicators. There was no difference between the intervention and control children on any anthropometric indicator (Table 2).

## 4 DISCUSSION

The main findings were that the cognitive scores of the intervention children were better than those of the control children, but language and motor development scores showed no overall difference. The cognitive effect size of 0.17 is equivalent to the difference in 4 IQ points (4 IQ points based on the standardized cognitive scores of 10.3 and 9.9 in the intervention vs. control communities, respectively). The cognitive effect size observed was lower than 0.48 obtained in a meta‐analysis of the two randomized trials where pregnant women were supplemented with iodine capsules and children assessed after birth (Bougma et al., 2013). Even though the mean IQ of the children in both groups were within normal, the 4 IQ points difference in the intervention community gives an advantage to adapt to their environment and learn compared with the controls. This is consistent with studies showing better mental development among children whose mothers were supplemented with iodine during pregnancy (Bougma et al., 2013).

At enrollment, mothers in the intervention group were expected to have had longer exposure to iodized salt than those in the control group. The intervention had improved the iodine status of the women by the time they were enrolled in this study. The difference is most likely due to intervention markets receiving iodized salt for a longer period of time and receiving a higher quality of iodized salt. One biological indicator supporting this conclusion is the median UIC level of the intervention pregnant women that was higher and indicated adequate iodine status although that from the control communities indicated, on average, mild ID (Delange, 2007). Child development can be affected even when their mothers are mildly iodine deficient in pregnancy (Berbel et al., 2009; Velasco et al., 2009). Consistent with the higher median UIE, there was a lower proportion of mothers with ID status in the intervention compared with the control villages. This implies that fewer children in the intervention villages were likely to have compromised development due to maternal ID during pregnancy. Finally, there was a higher goiter prevalence among control mothers suggesting a longer presence of ID in these women or their poorer iodine status prepregnancy and during pregnancy. The UI excretion and goiter indictors are consistent with there being higher levels of iodine intake and earlier exposure of intervention mothers to iodized salt. The intervention was therefore able to meet the iodine requirement of the pregnant women at the population level.

In contrast, none of the other mental development tests and growth was different between intervention and control groups. This may indicate that ID in the control communities (they also received iodized salt through market forces) was not severe enough to affect the other indicators of mental development and growth of the infants or that cognitive skills are more sensitive to iodine levels. Thus, the true effect of the iodized salt intervention may have been underestimated because of these group differences. Furthermore, children were very young and in infancy their language, particularly expressive language, may not be developed enough to show clear benefits from nutritional input. Supplementation of 200–300 μg potassium iodate in mild iodine deficient pregnant women did not see significant improvement in child language development (Berbel et al., 2009; Velasco et al., 2009). Also, low scores on the HOME inventory showed that psychosocial stimulation was equally low for both groups of children, thus impeding language development. Low psychosocial stimulation has been shown to be associated with language delay (Malhi, Sidhu, & Bharti, 2014). A combination of ID and severe iron deficiency anemia results in the reduction of heme‐dependent thyroperoxidase activity in the thyroid and might blunt the efficacy of iodine prophylaxes (Zimmermann, 2006; Zimmermann, Burgi, & Hurrell, 2007). A study in Cote d'Ivoire by Zimmermann, Adou, Torresani, Zeder, and Hurrell (2000) showed that an oral dose of iodized oil significantly improved iodine status in the nonanemic group compared with anemic group. Anemia, however, did not come out as a significant modifier of the effect of the intervention on cognition in this study. Despite the higher prevalence of anemia among mothers in pregnancy and their young children in the intervention communities compared with those of the control communities, intervention children's cognitive development remained higher than that of the control children. The effect on growth may have been masked by high incidence of growth restrictions in both communities (stunting 36%) making it difficult to observe any effect of iodine. There may also be greater underlying conditions such as their poor dietary diversity, low assets, poor water and sanitation, and high morbidity, which may affect their growth more than the intervention. Our data are consistent with other supplementation trials, such as the Peruvian study that reported no significant difference in the gross motor development and child growth rate between the intervention and control communities (Pretell et al., 1972). A meta‐analysis of iodine intervention studies in pregnancy has shown no effect on gross motor development and growth of children (Zhou, Anderson, Gibson, & Makrides, 2013).

The effect of iodine on child cognition was modified by maternal depression, child psychosocial stimulation (HOME score), and maternal education. The quality of home stimulation or additional learning opportunities by itself has been shown to be associated with increased child cognitive development by an effect size of 0.5 to 1.0 (Walker et al., 2007). The intervention gave a mean difference in IQ of 4 between the groups, and this increased to an IQ difference of 9 in low stimulation. The evidence supports the importance of early cognitive stimulation for a child to reach his/her developmental potential. Here, the improvement of child cognition was observed only in children from households that scored low on psychosocial stimulation, suggesting that iodized salt intake compensated for the low stimulation. In contrast, the modifier effects of maternal well‐being (reflected in low depressive symptoms) and maternal education indicated that iodine raised mental development only when these resources were additionally available to the child.

Some limitations of the study exist. First, the control children were also receiving iodized salt by the endline data collection. Consequently, there was no control group with very low levels of iodized salt, as experienced during baseline assessment in the broader study. Second, there was no assessment of iodine status of mothers throughout their pregnancy to find out when they became iodine sufficient. Mothers were not recruited and assessed at the same stage of pregnancy and children were measured at different ages between 2 and 13 months of age. Consequently, this meant that children had different exposures to iodized salt during fetal development, during lactation, and during complementary feeding.

## 5 CONCLUSION

This iodized salt intervention improved the iodine status of pregnant women and their children. The cognition of the infants and young children in the intervention communities was higher than those in the control communities, indicating exposure to iodized salt from early pregnancy to infancy resulted in higher cognitive development.

## CONFLICTS OF INTEREST

The authors declare that they have no conflicts of interest.

## CONTRIBUTIONS

HM was responsible for the design of study, field data collection, data analysis and interpretation, manuscript preparation, and has full responsibility for the final manuscript. GM and FA were responsible for the conception, design, overview of the study, overview of data analysis and interpretation, and contributed to the final manuscript. KBH contributed to project oversight and editing of final paper. AS was responsible for the laboratory analysis at EPHI, local coordination of project activities with Ethiopian institutions, and reviewed the presentations from the project.
